# Bipolar disorder and the gut microbiota: a bibliometric analysis

**DOI:** 10.3389/fnins.2024.1290826

**Published:** 2024-03-21

**Authors:** Xiaoxiao Lin, Jinyu Huang, Shuai Wang, Kai Zhang

**Affiliations:** ^1^Hangzhou First People’s Hospital, Hangzhou, China; ^2^Department of Psychiatry, Chaohu Hospital of Anhui Medical University, Hefei, China; ^3^School of Mental Health and Psychological Sciences, Anhui Medical University, Hefei, China

**Keywords:** bipolar disorder, gut microbiota, a bibliometric analysis, gut-brain axis, interventions

## Abstract

**Background:**

Previous studies have explored the relationship between bipolar disorder and gut microbiota. However, there has been no bibliometric analysis to summarize and analyze these publications. Our objective was to perform a bibliometric analysis to investigate the current status and frontiers of the publications in the field of the association between bipolar disorder and the gut microbiota.

**Methods:**

We retrieved publications concerning the interplay between the gut microbiota and bipolar disorder from the Web of Science Core Collection (WoSCC). The analysis was executed using WoSCC’s literature analysis tool and VOSviewer 1.6.16.

**Results:**

In total, we identified 177 publications originating from 362 institutions across 39 countries/regions, and these articles were disseminated in 104 different journals. The most productive institutions, authors, countries/regions, and journals were Zhejiang University contributing 18 publications, Shaohua Hu authoring 12 publications, China with 53 publications, and *Frontiers in Psychiatry* with 11 publications. The first high-cited document was published in the Journal of Psychiatric Research in 2017, and authored by Evans. In this article, they found gut microbiome composition was associated with BD and its illness severity, and they concluded that targeting the gut microbiota may be helpful to develop the effective treatment for bipolar disorder. The top 5 keywords with the highest frequency except for bipolar disorder and gut microbiota were as follows: depression, inflammation, probiotic, gut-brain axis, and anxiety.

**Conclusion:**

In conclusion, this is the first bibliometric analysis to explore the publications in the field of the association between bipolar disorder and the gut microbiota. The main research hotspots regarding this field were the characteristics, abundance, and diversity of gut microbiome in bipolar disorder, the role of treatment and gut microbiome in bipolar disorder, microbiome-brain connections in bipolar disorder, and interventions for bipolar disorder based on microbiota composition modification. The number of studies about the association between gut microbiota and bipolar disorder is relatively small, and more studies are needed to expand our understanding the association between gut microbiota and bipolar disorder.

## Introduction

Bipolar disorder is a severe psychiatric disorder which is characterized by hypomanic states, manic episodes, and the interweaving or alternating occurrences of depressive episodes (Craddock and Sklar, 2013; Grande et al., 2016; Scott et al., 2017; Carvalho et al., 2020; McIntyre et al., 2020; Goes, 2023). It is estimated that the 12-month and lifetime prevalence for bipolar disorder is 1.5 and 2.4%, respectively. It was reported 6 to 7% of patients with bipolar disorder committed suicide (Carvalho et al., 2020). The treatment for bipolar disorder consists two aspects of acute management and long-term management (Goes, 2023). In the phase of acute management, antipsychotics and mood stabilizers are the mainstay of depression and bipolar mania. In addition, electroconvulsive therapy (ECT) is effective for treatment-resistant patients, particularly those with catatonic features or psychotic. For the phase of long-term management, the combinations of pharmacological, psychological, and lifestyle interventions should be used (Carvalho et al., 2020). More strategies should be explored to prevent and treat the bipolar disorder.

In recent years, the studies about bipolar disorder and the gut microbiome become more and more popular (Evans et al., 2017; Zheng et al., 2020; Li et al., 2022). The gut microbiota is a rapidly advancing biomedical frontier, which is associated with psychiatric diseases including schizophrenia (Samochowiec and Misiak, 2021; Yang et al., 2022), bipolar disorder (Li et al., 2022), depression (Zhu et al., 2021; Liu et al., 2023), and autism (Sharon et al., 2019; Lin et al., 2022). Gut microbiota, act as “metabolic machinery,” can influence many aspects of physiology through immunological hormonal, and neural pathways (Hu et al., 2019). The gut microbiota can influence host metabolism, and interact with the central nervous system by gut-brain axis (Basiji et al., 2023). Gut microbiota may influence the pathophysiology and etiology of bipolar disorder by disrupting homeostatic regulation (Li et al., 2022). Understanding the association between gut microbiota in bipolar disorder (BD) will be helpful to find new effective disease markers and treatment strategies (Fond et al., 2015; Nguyen et al., 2018). Bibliometric analysis is the most frequently used method to summarize the current status and predict developmental trends by analysis of many components such as authors, countries, institutions, and citations for overall studies in a specific field. By this method, previous studies have explored the association between gut microbiota and many psychiatric conditions including schizophrenia (Yang et al., 2022), depression (Zhu et al., 2021), and autism (Lin et al., 2022). However, there was no bibliometric study to explore the association between gut microbiota and bipolar disorder. Bibliometric analysis is a methodological approach used to quantitatively assess academic literature, providing a comprehensive overview of research trends, patterns, and networks in a specific field. It involves statistical analysis of various aspects of scientific publications, such as publication and citation counts, to gauge the impact and influence of research works, authors, or journals. This analysis also includes examining the relationships and collaborations between authors, institutions, and countries, as well as identifying dominant themes, emerging trends, and potential gaps in the research through content analysis. Conducting a bibliometric analysis of the relationship between BD and gut microbiota is crucial can provide a comprehensive overview of the research landscape, identifying pivotal studies, trends, and gaps in this emerging field. Therefore, our study aims to conduct a bibliometric analysis for the publications in the field of the association between gut microbiota and bipolar disorder, to determine the current status and frontiers in this field.

## Materials and methods

In our study, all relevant publications were extracted from Web of Science Core Collection (WoSCC). The search terms were: (microbiome* OR microbiot* OR microflora OR microbiota OR microbiome) AND (intestin* OR intestinal OR gut OR gastro-intestin* OR gastrointestinal*) AND (“bipolar disorder” or “bipolar depression” or “bipolar disorders” or mania or “mood disorder” or “affective disorder” or “mood disorders” or “affective disorders”) from inception to 1 June 2023. Two reviewers (Lin and Wang) investigated each study according to inclusion criteria by screening the titles, abstracts, and full-text of publications. They discussed with the third author (Huang) if they could not reach an agreement about some publications.

After the selection process, we downloaded and imported the TXT format containing “Full Record and Cited References” into the VOSviewer software. Our analysis encompassed two stages, utilizing both the WoSCC literature analysis system and the VOSviewer 1.6.16 software. The WoSCC literature analysis system was used to analysis categories, publication years, document types, *h*-index and the distribution of institutions, countries/regions and authors. We summarized the leading 10 authors, institutions, countries/regions, and journals in terms of the number of publications, and provided the information of the 20 most highly cited publications. VOSviewer1.6.16 software was used to perform co-authorship of authors, organizations, and countries, co-occurrence of all keywords and co-citation of cited references. For co-occurrence analysis of keywords, we merge the synonyms of “gut microbiota,” “gut microbiome,” “fecal microbiota,” “gut-microbiota,” “intestinal microbiota,” “microbiota,” and “microbiome” into the term “gut microbiota”; “major depressive disorder” and “depression” into the term “depression.”

## Results

### Literature search and trends analysis

**FIGURE 1:**
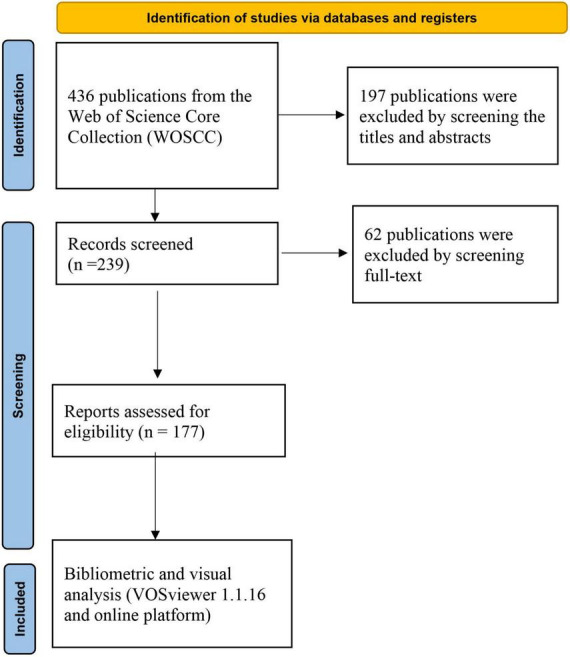
Search flowchart.

**FIGURE 2:**
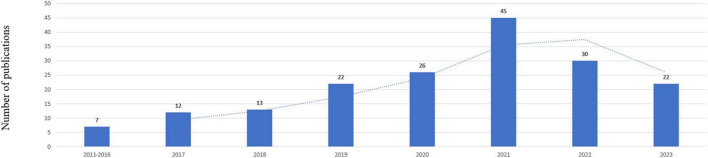
Annual publications quantitative distribution.

A total of 436 records were yielded in our primary database search. After screening titles, abstracts, and full text, a total of 177 publications were included in the analysis, which was shown in Figure 1. The total number of publications per year was listed in Figure 2. The documents can be classified into two phases: documents published before 2017 were in the first phase. In this phase, the number of documents was small annually. The second phase was 2017–2023, and in this phase, the number of documents was all beyond 10 publications per year. For subject area of documents, the top two subject categories were the psychiatry and neurosciences, accounting for 79 and 56 publications, respectively.

### Country/region, institution and author analysis

**FIGURE 3:**
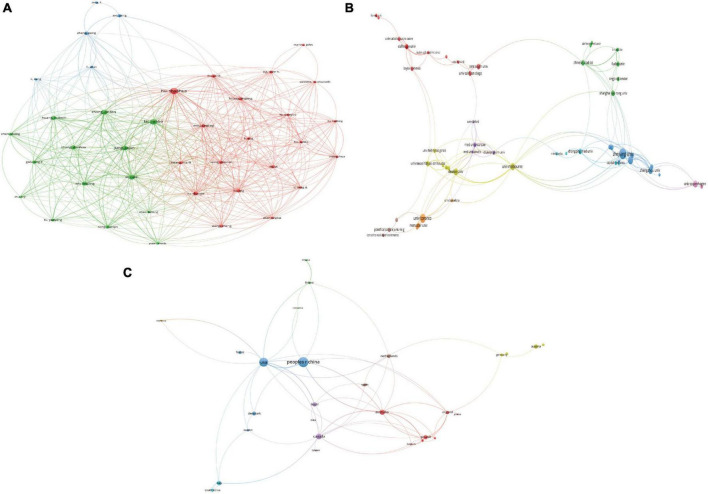
Visualization knowledge maps of authors, institutions, and countries/regions **(A)** The co-authorship map of authors **(B)** the co-authorship map of institutions **(C)** the co-authorship map of countries/regions.

In total, 394 institutions across 46 countries/regions were included. The most productive institution was Zhejiang University contributing 18 publications, which was followed by University of Toronto contributing 11 publications, and Baylor College of Medicine contributing 9 publications. The top 3 productive countries were China with 53 publications, USA with 42 publications, and Canada with 19 publications. For most productive authors, Shaohua Hu contributed the most publications with 12 documents, and Peifen Zhang, Caixi Xi, Jianbo Lai with 9 publications following him. The top 10 most productive organizations, authors, and countries/regions were shown in Table 1. In addition, the network visualization maps of co-authorship of countries, authors, and institutions were displayed in Figure 3, and the top 3 cooperative authors were Shaohua Hu (TLS = 118), Jianbo Lai (TLS = 113), and Caixi Xi (TLS = 89). For organizations, they were Zhejiang University (TLS = 47), Melbourne University (TLS = 30), and Deakin University (TLS = 30). For countries/regions, they were USA (TLS = 34), Australia (TLS = 25) and Canada (TLS = 22).

**TABLE 1:** The top 10 productive authors, institutions and countries based on publications.

Ranking	Country	Number	Citations	H-index
**Publications**				
1	China	53	897	17
2	USA	42	1,607	20
3	Canada	18	404	10
4	Australia	15	520	11
5	Austria	10	250	6
6	Brazil	10	268	7
7	Italy	10	93	5
8	Poland	9	255	8
9	Denmark	8	197	7
10	Netherlands	8	233	7
1	Zhejiang University	18	421	8
2	University of Toronto	11	284	7
3	Baylor College of Medicine	9	173	5
4	University of Texas System	9	213	6
5	University of Copenhagen	8	197	7
6	University of Melbourne	8	382	6
7	University of Michigan	8	377	4
8	Chinese Academy of Sciences	7	148	5
9	Deakin University	7	307	6
10	Medical University of Graz	7	189	4
1	Shaohua Hu	12	284	6
2	Pei fen Zhang	9	130	5
3	Caixi Xi	9	94	5
4	Jianbo Lai	9	130	5
5	Jiajun Jiang	8	94	5
6	Sabrina Moerkl	7	188	4
7	E. Z Reininghaus	6	219	5
8	Martina Platzer	6	197	4
9	Nina Dalkner	6	219	5
10	Antonio L Teixeira	6	185	6

### Journal and high-cited publication analysis

**FIGURE 4:**
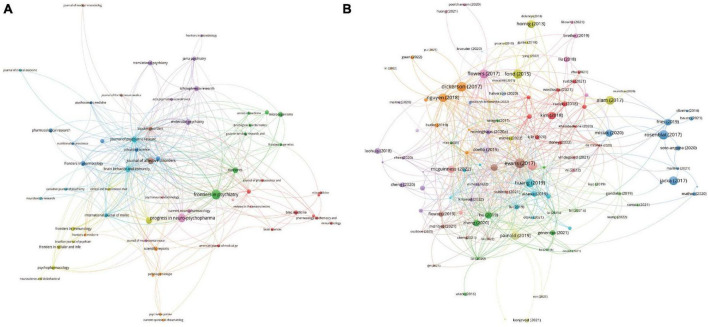
Visualization knowledge maps of journals and references **(A)** citation of journal; **(B)** citation of references.

**FIGURE 5:**
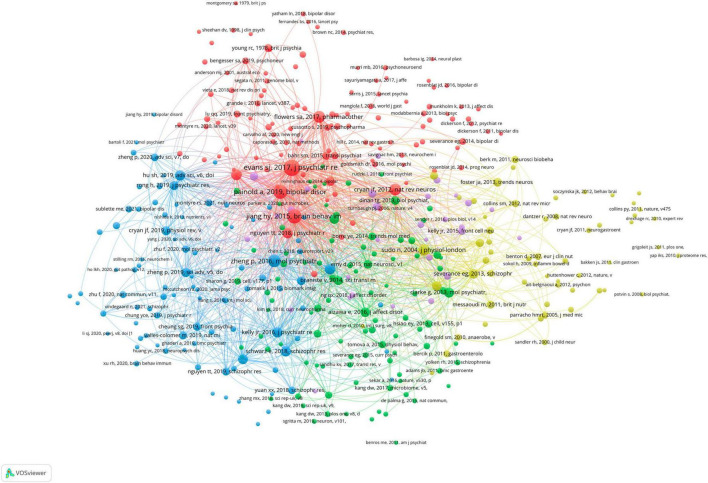
The network visualization map of co-citation of references.

All publications were distributed in 112 journals. Among them, *Frontiers in Psychiatry* was the most productive journal with 11 publications, and followed by *Progress in Neuro-Psychopharmacology & Biological Psychiatry* with 8 publications, and *Journal of Affective Disorders* with 5 publications. The top 10 most productive journals were summarized in Table 2. For the citation of publications, the first high-cited document was in *Journal of Psychiatric Research* in 2017, and authored by Evans et al. (2017). In this study, 115 patients with bipolar disorder and 64 control subjects were included and the stool microbiome were measured by the stool microbiome. They found gut microbiome compositions were associated with BD and its illness severity, and they concluded that targeting the gut microbiota may be helpful to develop effective treatment for bipolar disorder. The second high-cited document was in *Brain Behavior and Immunity* in 2017, and authored by Dickerson et al. (2017). In their article, they believed that the successful development of therapeutic agents which can alter gut microbiome and gastrointestinal inflammation in bipolar disorder will be helpful to develop novel effective strategies to prevent and treat it. The third high-cited document was in *JAMA Psychiatry* in 2021, and authored by Nikolova et al. (2021). In this article, they found that gut microbiome perturbations were related to the transdiagnostic pattern with bipolar disorder. The characteristics of top 20 most high-cited publications (Hornig, 2013; Fond et al., 2015; Alam et al., 2017; Dickerson et al., 2017; Evans et al., 2017; Flowers et al., 2017; Jacka, 2017; Rosenblat and McIntyre, 2017; Kim and Shin, 2018; Nguyen et al., 2018; Coello et al., 2019; Fries et al., 2019; Hu et al., 2019; Huang et al., 2019; Painold et al., 2019; Rong et al., 2019; Misiak et al., 2020; Zheng et al., 2020; Nikolova et al., 2021; McGuinness et al., 2022) were displayed in Table 3. The network visualization maps of citations of journals and documents were summarized in Figure 4. In addition, Figure 5 displayed the co-cited references, which are cited by more than one article in these 177 documents, and the top 3 co-cited references were consistent with the top three high-cited publications.

**TABLE 2:** The top 10 productive journals based on publications.

Ranking	Journal name	Country	Counts	Citation
1	*Frontiers in Psychiatry*	Switzerland	11	235
2	*Progress in Neuro-Psychopharmacology & Biological Psychiatry*	England	8	197
3	*Journal of Affective Disorders*	Netherlands	5	50
4	*Neuro-psychobiology*	Switzerland	5	85
5	*Brain Behavior and Immunity*	USA	4	258
6	*Journal of Psychiatric Research*	England	4	373
7	*Molecular Psychiatry*	England	4	144
8	*Nutrients*	Switzerland	4	61
9	*CNS Neuroscience Therapeutics*	China	3	5
10	*Frontiers in Pharmacology*	Switzerland	3	23

**TABLE 3:** The top 20 most high-cited references.

Rank	Title	Journal	Total citations	Year	First author
1	The gut microbiome composition associates with bipolar disorder and illness severity	*Journal of Psychiatric Research*	161	2017	Simon Evans
2	The microbiome, immunity, and schizophrenia and bipolar disorder	*Brain Behavior and Immunity*	155	2017	Faith Dickerson
3	Perturbations in gut microbiota composition in psychiatric disorders a review and meta-analysis	*JAMA Psychiatry*	146	2021	Yang Yang
4	Bipolar disorder and immune dysfunction: epidemiological findings, proposed pathophysiology and clinical implications	*Brain Sciences*	139	2017	Gregory H. Jones
5	The “psychomicrobiotic”: targeting microbiota in major psychiatric disorders: a systematic review	*Pathologie Biologie*	139	2015	Duygu Agagunduz
6	Interaction between atypical antipsychotics and the gut microbiome in a bipolar disease cohort	*Pathologie Biologie*	137	2017	Li Huang
7	Microbiome, inflammation, epigenetic alterations, and mental diseases	*American Journal of Medical Genetics Part B-Neuropsychiatric Genetics*	126	2017	Ting-Ting Huang
8	Nutritional psychiatry: where to next?	*Ebiomedicine*	121	2017	Crystal Obi-Azuike
9	Current understanding of gut microbiota in mood disorders: an update of human studies	*Frontiers in Genetics*	117	2019	Cherise R. Chin Fatt
10	Overview and systematic review of studies of microbiome in schizophrenia and bipolar disorder	*Journal of Psychiatric Research*	117	2018	Jianzhao Zhang
11	A step ahead: exploring the gut microbiota in inpatients with bipolar disorder during a depressive episode	*Bipolar Disorders*	104	2019	Kan Yu
12	The microbiota-gut-brain axis in neuropsychiatric disorders: patho-physiological mechanisms and novel treatments	*Current Neuropharmacology*	103	2018	Andrea Schneider
13	The role of microbes and autoimmunity in the pathogenesis of neuropsychiatric illness	*Current Opinion in Rheumatology*	99	2013	Hamid Mostafavi Abdolmaleky
14	The HPA axis dysregulation in severe mental illness: can we shift the blame to gut microbiota?	*Progress in Neuro-Psychopharmacology & Biological Psychiatry*	86	2020	Wujie Ye
15	A systematic review of gut microbiota composition in observational studies of major depressive disorder, bipolar disorder and schizophrenia	*Molecular Psychiatry*	83	2022	Ilya Smolensky
16	Gut microbiota changes in patients with bipolar depression	*Advanced Science*	83	2019	Antonina Kurowska
17	Similarly in depression, nuances of gut microbiota: evidences from a shotgun metagenomics sequencing study on major depressive disorder versus bipolar disorder with current major depressive episode patients	*Journal of Psychiatric Research*	80	2019	James Melrose
18	Gut microbiota composition in patients with newly diagnosed bipolar disorder and their unaffected first-degree relatives	*Brain Behavior and Immunity*	79	2019	Weiming Gong
19	Revisiting inflammation in bipolar disorder	*Pharmacology Biochemistry and Behavior*	73	2019	Yaning Zang
20	Gut microbial signatures can discriminate unipolar from bipolar depression	*Advanced Science*	72	2020	Peifen Zhang

### Keyword analysis

**FIGURE 6:**
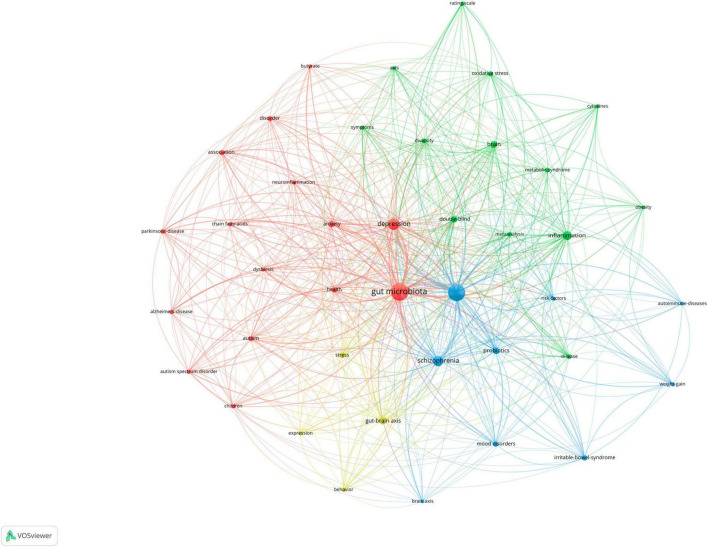
Visualization of keyword co-occurrence analysis.

The map of the co-occurrence of keywords was displayed in Figure 6, and there were four research directions are also shown. The green cluster includes inflammation and brain. The red cluster includes gut microbiota, depression, and anxiety. The blue cluster includes bipolar disorder, schizophrenia, and probiotic. The yellow cluster includes gut-brain axis and stress. The top 5 keywords with the highest frequency except for bipolar disorder and gut microbiota were as follows: depression (*N* = 62), schizophrenia (*n* = 50), inflammation (*N* = 39), gut-brain axis (*N* = 25), and brain (*N* = 24).

## Discussion

### General information

To the best of knowledge, it is the first bibliometric analysis of publications in the field of bipolar disorder and the gut microbiome. There were 177 documents from 362 organizations across 39 countries/regions, which were published in 104 journals. The most productive organizations, authors, countries/regions, and journals were Zhejiang University contributing 18 publications, Shaohua Hu authoring 12 publications, China with 53 publications, and *Frontiers in Psychiatry* with 11 publications. The first high-cited document was in *Journal of Psychiatric Research* in 2017, and authored by Evans et al. (2017). In this article, they found gut microbiome composition were associated with BD and its illness severity, and they concluded that targeting the gut microbiota may be helpful to develop effective treatment for bipolar disorder. The top 5 keywords with the highest frequency except for bipolar disorder and gut microbiota were as follows: depression, inflammation, probiotic, gut-brain axis, and anxiety. Most of the included publications were from China and USA (95/177, 53.7%), and the number of publications from other countries/regions should be improved.

### Hotspots and frontiers

According to top 20 most high-cited documents and the core keywords, the research hotspots and frontiers were summarized as follows:

(1) The characteristics, abundance, and diversity of gut microbiome in bipolar disorder. In the top 20 most high-cited references, 11 publications (Evans et al., 2017; Flowers et al., 2017; Nguyen et al., 2018; Coello et al., 2019; Hu et al., 2019; Huang et al., 2019; Painold et al., 2019; Rong et al., 2019; Zheng et al., 2020; Nikolova et al., 2021; McGuinness et al., 2022) explored the characteristics abundance, and diversity of gut microbiome in bipolar disorder. Some systematic review and meta-analysis (Nguyen et al., 2021; Sublette et al., 2021; Vindegaard et al., 2021; McGuinness et al., 2022; Obi-Azuike et al., 2023) explored this topic, and they were summarized in Table 4. More and more studies have demonstrated disparities in gut microbiome, abundance, and diversity in BD (Chang et al., 2014; Evans et al., 2017; Flowers et al., 2017; Bengesser et al., 2018; Aizawa et al., 2019; Coello et al., 2019; Hu et al., 2019; Jiang et al., 2019; Lu et al., 2019; Painold et al., 2019; Rong et al., 2019; Vinberg et al., 2019; Lai et al., 2021; McIntyre et al., 2021). According to these systematic reviews (Nguyen et al., 2021; Sublette et al., 2021; Vindegaard et al., 2021; McGuinness et al., 2022; Obi-Azuike et al., 2023), it may be confirmed that there were reductions in overall microbial richness in BD. The findings about the α-diversity metrics including microbial richness were controversial. It seems that alterations in *Ruminococcaceae*, *Faecalibacterium*, *Actinobacteria*, *Ruminococcus*, *Lachnospiraceae*, and *Bacteroidetes* were most represented. In addition, the causation of alterations in the gut microbiota and bipolar disorder cannot be determined based on current studies since most of them are observational.

**TABLE 4:** The systematic review and meta-analysis of the characteristics, abundance, and diversity of gut microbiome in bipolar disorder.

References	Included studies of BD	Included participants	The main findings
Obi-Azuike et al., 2023	12 articles	613 BD patients	There was overall difference in gut microbiota composition, but the alterations found were not consistent. Differences in *Lactobacillus*, *Faecalibacterium*, and *Ruminococcus* abundance was found to be the most consistent. Probiotic supplementation can lower patient rehospitalizations and improve cognitive impairments and depressive symptoms significantly.
Vindegaard et al., 2021	4 studies	299 BD cases and 209 non-BD controls	There was higher abundance of *Actinobacteria*, and lower abundance of *Firmicutes*, *Lachnospiraceae*, *Faecalibacterium*
Nguyen et al., 2021	7 studies	520 BD cases	*Ruminococcaceae* and *Faecalibacterium* were relatively decreased in BD
Sublette et al., 2021	13 studies	474 patients with BD and 285 non-BD controls	The low α-diversity and dysbiosis of abundance of *Faecalibacterium* and *Bacteroides* may characterize BD
McGuinness et al., 2022	7 studies	527 BD cases and 477 non-BD controls	There were differences in overall community composition (β-diversity), but no strong evidence for a difference in the number or distribution (α-diversity)

(2) The role of treatment and gut microbiome in bipolar disorder. A few studies explored the association between medication treatment for bipolar disorder and gut microbiome. For example, Flowers et al. (2017) found that the treatment of atypical antipsychotic (AAP) was associated with specific representation of gut bacterial families, and it is also is related to decreased species richness in female. Lai et al. (2022) focused on understanding the gut microbiota changes in bipolar disorder (BD) patients experiencing depressive episodes and the effects of quetiapine monotherapy. The research involved 62 BD patients and 60 healthy individuals, with fecal samples collected for metagenomic sequencing. The study found that BD patients had specific alterations in gut microbial diversity and composition, which were notably modified after 1 month of quetiapine treatment. A significant finding was the correlation of *Clostridium bartlettii* abundance with factors like patient age, baseline depression severity, and brain function, particularly in the hippocampus. The study also developed random forest models based on bacterial species, achieving reasonable accuracy in distinguishing between patients and controls, and between treatment responders and non-responders. These results suggest that gut microbiota alterations could serve as potential biomarkers for diagnosing BD and predicting treatment outcomes.

(3) Microbiome-brain connections in bipolar disorder. In the top 20 most high-cited references, 7 publications (Hornig, 2013; Alam et al., 2017; Dickerson et al., 2017; Rosenblat and McIntyre, 2017; Kim and Shin, 2018; Fries et al., 2019; Misiak et al., 2020) explored the microbiome-brain connections in bipolar disorder. For core keywords, inflammation in the green cluster, and gut-brain axis in the yellow cluster were related to this topic. Microbiome-brain connections in bipolar disorder may be associated with inflammation (Yuan et al., 2019; Jones et al., 2021; Lai et al., 2021), tryptophan metabolism (Barbuti et al., 2017; Wang and Miller, 2018; Wang et al., 2019), microglia (Cenit et al., 2017; Morais et al., 2021), the aryl hydrocarbon receptor (Reiter et al., 2018; Neamah et al., 2020; Tischkau, 2020; Barroso et al., 2021), and endocrine function (Perry et al., 2019; Anderson and Maes, 2020). A recent study (Li et al., 2022) used multi-omics analyses to explore microbiome-brain connections, and in this study, 109 unmedicated patients with BD and 40 controls were included. The serum metabolomics, fecal metagenomic, and neuroimaging were used to explore the characteristics of microbial-gut-brain axis in BD. The findings revealed the identification of BD-associated neuroactive microbes and metabolites, which emerged as potential markers linked to distinct features of brain network functional connectivity in BD. These markers suggested possible implications for disrupted cognitive functioning and emotional regulation. In a comprehensive analysis of over 12,000 measured metabolic features, a substantial divergence (73.54%) in serum metabolome profiles was observed between BD patients and healthy controls. This divergence pinpointed distinctively abundant microbial-derived neuroactive metabolites, encompassing various gamma-aminobutyric acid, kynurenic acid, B vitamins, and short-chain fatty acids. These identified metabolites demonstrated potential connections with the prevalence of specific gut microbiota species, each with corresponding biosynthetic capabilities. Notable among them were *Akkermansia muciniphila*, *Citrobacter* spp, *Phascolarctobacterium* spp, *Yersinia* spp, *Enterobacter* spp, and *Flavobacterium* spp. Some bacteria may play an important role as “psychobiotic” in the gut-brain axis connection between bipolar disorder and gut microbiome. This approach unveiled possible signaling pathways connecting the gut, the microbiome, and the brain, suggesting a potential contribution to the underlying mechanisms of BD.

(4) Interventions for bipolar disorder based on microbiota composition modification. In the top 20 most high-cited references, 5 publications mentioned the microbial-based interventions for bipolar disorder (Fond et al., 2015; Dickerson et al., 2017; Evans et al., 2017; Jacka, 2017; Rosenblat and McIntyre, 2017). For core words, the probiotic in the blue cluster was related to this topic. It may be a promising avenue to combine microbial-based interventions and standard therapy for improving certain parameters like hospitalization length, cognition, and metabolic side effects in bipolar disorder. For example, Reininghaus et al. (2018) conducted pilot study to analyze the effect of probiotic supplements on cognitive parameters in patients with bipolar disorder, and found that probiotic supplement might be helpful to improve the cognitive function in patients with bipolar disorder. Dickerson et al. (2018) conducted a trial to investigate whether the administration of probiotic supplement can prevent psychiatric rehospitalizations in individuals with BD, and in their study, 66 patients with BD were randomized to receive 6 months of adjunctive probiotics or adjunctive placebo. The results demonstrated that 8 rehospitalizations in the 33 patients who received the probiotics while 24 rehospitalizations in the 33 patients who received placebo (*P* = 0.009), and what’s more, probiotics was associated with a significant advantage (*P* = 0.007). They concluded that Probiotic supplementation may be associated with a lower rate of rehospitalization. A systematic review (Sublette et al., 2021) demonstrated that probiotic supplementation can lower patient rehospitalizations and improve cognitive impairments and depressive symptoms significantly in patients with BD. In addition, fecal microbial transplantation (FMT) may be a potentially effective strategy for the treatment of bipolar disorder.

Our study has some limitations that should be acknowledged. Firstly, we solely utilized the WoSCC database due to the constraints of the VOSviewer software, which prevented the analysis and visualization of co-citation maps from other databases such as Embase and PubMed. Secondly, the inclusion of publications from the year 2023 was incomplete due to our study’s cutoff date. In addition, there is a distinct role of gender in bipolar disorder. However, only few studies explored the gender differences between bipolar disorder and the gut microbiota (Flowers et al., 2019; Nikolova et al., 2021). Furthermore, the total number of publications in this specific domain remains relatively small, underscoring the necessity for further investigations to expand our understanding of the association between gut microbiota and bipolar disorder.

In conclusion, this is the first bibliometric analysis to investigate the publications in the field of the association between bipolar disorder and the gut microbiota. The main research hotspots regarding this field were the characteristics, abundance, and diversity of gut microbiome in bipolar disorder, the role of treatment and gut microbiome in bipolar disorder, microbiome-brain connections in bipolar disorder, and interventions for bipolar disorder based on microbiota composition modification. The number of studies about the association between gut microbiota and bipolar disorder is relatively small, and more studies are needed to expand our understanding of the link between gut microbiota and bipolar disorder.

## Data availability statement

The original contributions presented in this study are included in this article/supplementary material, further inquiries can be directed to the corresponding authors.

## Author contributions

XL: Data curation, Formal Analysis, Methodology, Project administration, Supervision, Writing – original draft. SW: Data curation, Funding acquisition, Methodology, Resources, Supervision, Writing – original draft, Writing – review & editing. JH: Formal Analysis, Funding acquisition, Project administration, Resources, Visualization, Writing – original draft, Writing – review & editing. KZ: Data curation, Supervision, Writing – original draft, Writing – review & editing.
